# Deep-learning-based in-field citrus fruit detection and tracking

**DOI:** 10.1093/hr/uhac003

**Published:** 2022-02-11

**Authors:** Wenli Zhang, Jiaqi Wang, Yuxin Liu, Kaizhen Chen, Huibin Li, Yulin Duan, Wenbin Wu, Yun Shi, Wei Guo

**Affiliations:** 1 Information department, Beijing University of Technology, Beijing, 100022, China; 2 Institute of Agricultural Resources and Regional Planning, Chinese Academy of Agricultural Sciences, Beijing 100081, China; 3 International Field Phenomics Research Laboratory, Institute for Sustainable Agro-ecosystem Services, The University of Tokyo, Tokyo 188-0002, Japan

## Abstract

Fruit yield estimation is crucial for establishing fruit harvest and marketing strategies. Recently, computer vision and deep learning techniques have been used to estimate citrus fruit yield and have exhibited notable fruit detection ability. However, computer-vision-based citrus fruit counting has two key limitations: inconsistent fruit detection accuracy and double-counting of the same fruit. Using oranges as the experimental material, this paper proposes a deep-learning-based orange counting algorithm using video sequences to help overcome these problems. The algorithm consists of two sub-algorithms, OrangeYolo for fruit detection and OrangeSort for fruit tracking. The OrangeYolo backbone network is partially based on the YOLOv3 algorithm, which has been improved upon to detect small objects (fruits) at multiple scales. The network structure was adjusted to detect small-scale targets while enabling multiscale target detection. A channel attention and spatial attention multiscale fusion module was introduced to fuse the semantic features of the deep network with the shallow textural detail features. OrangeYolo can achieve mean Average Precision (mAP) values of 0.957 in the citrus dataset, higher than the 0.905, 0.911, and 0.917 achieved with the YOLOv3, YOLOv4, and YOLOv5 algorithms. OrangeSort was designed to alleviate the double-counting problem associated with occluded fruits. A specific tracking region counting strategy and tracking algorithm based on motion displacement estimation were established. Six video sequences taken from two fields containing 22 trees were used as the validation dataset. The proposed method showed better performance (Mean Absolute Error (MAE) = 0.081, Standard Deviation (SD) = 0.08) than video-based manual counting and produced more accurate results than the existing standards Sort and DeepSort (MAE = 0.45 and 1.212; SD = 0.4741 and 1.3975).

## Introduction

With the introduction of computer vision in the smart agriculture domain, fruit detection and tracking technologies, which can help to obtain fruit yield statistics and assist with automatic fruit picking and automated orchard management, have become important areas of research. Anderson et al. [1] pointed out that yield prediction can help growers with farming decisions for the season, especially in regard to labor allocation for harvesting, fruit transportation, and storage methods. An experienced agronomist can use these resources and combine this information with knowledge of fruit tree physiology to provide advice on the current season and make recommendations on management practices for optimizing orchard development. Anderson et al. [1] also pointed out that fruit yield prediction can now be broadly classified into five methods. Fruit yield estimation is a labor-intensive, monotonous, and tedious task. However, the use of computer vision techniques can quickly detect fruits and thus predict the overall yield of an orchard, and fruit yield prediction methods based on computer vision with deep learning have shown considerable potential in recent years [2]. Nonetheless, computer-vision-based fruit counting has two limitations: inconsistent fruit detection and double-counting of the same fruit.

To solve the problem of fruit detection accuracy, many researchers have proposed deep-learning-based detection algorithms. Fruit detection algorithms can be broadly classified into detection based [3–10] and segmentation based [11–14] algorithms. However, fruits appear at multiple scales, and it is difficult to extract the effective features from both small- and large-scale fruits in order to detect fruits accurately. Although existing techniques can effectively enhance the accuracy of fruit detection, the presence of small-scale targets has not been considered. In particular, Mu et al. [5] developed a tomato detection model based on a faster region-based convolutional neural network (R-CNN) with Resnet-101 and deep learning approaches to automatically detect intact green tomatoes, regardless of fruit growth stage or the presence of occlusions. Detection on the test dataset corresponded to an average precision of 87.83% (intersection over union ≥0.5). The authors reported that large tomatoes tended to have high scores, whereas some small tomatoes had scores of less than 0.2. Jordi [13] used a mask R-CNN instance segmentation neural network to detect apples, and the results showed that some small apples were difficult to detect. Existing techniques typically ignore the problem of small fruit size and severely overlapping fruits. Therefore, although existing algorithms can achieve high-precision fruit detection in scenarios with simple backgrounds and few fruit targets, it is difficult to achieve satisfactory results in actual orchards because the fruit sizes tend to be variable. In addition, some researchers have found that matching the perceptual field to the target scale can improve the detection accuracy for small targets [25, 26]. The Receptive Field is the area size of the pixel points on the output feature map from each layer of the convolutional neural network mapped on the input image. The attention mechanism can enhance the effective information, such as the weight of features, and reduce the influence of invalid information, thereby enhancing the performance of computer vision tasks [35, 36]. This approach can be adopted for fruit detection applications to improve detection accuracy.

To avoid double-counting of the same fruit, some researchers have proposed solutions based on static images [3, 4, 11, 12, 15, 16] and video sequences [17–23]. The video-sequence-based counting method, in which fruit images are collected from multiple viewpoints, thereby enabling the observation of more fruit, shows promise for enabling fruit counting algorithms to be used in actual orchard fields. Multi-objective tracking algorithms are the key to solving the counting problem. Among them, Sort [28] and DeepSort [29] combine the Kalman filter and Hungarian algorithm to accomplish the multi-target tracking task, and they have a wide range of applications in the field of foot traffic counting. Although these methods have been validated in the MOT dataset [38] and have yielded satisfactory tracking results, they do not work well for fruit counting. The main reason is the dense fruit growth and heavy fruit overlap in real orchards. Notably, Wang et al. [17] combined MangoYOLO and the Hungarian algorithm to track and count mangoes in a video of mango trees. They demonstrated experimentally that the counting method based on video sequences was considerably more accurate than counting based on static images [3]. In addition, some researchers have used 3D techniques for counting. For example, Gan et al. [23] used the Global Navigation Satellite System, an inertial measurement unit (IMU), and LIDAR to achieve simultaneous localization and used an extended Kalman filter to improve the reliability and accuracy of the localization. However, the 3D counting method requires expensive equipment, and the system is highly complex, making it difficult to obtain useful 3D reconstruction results.

In summary, the counting method based on video sequences is an effective and economical solution that can be successfully implemented. However, in actual orchards, oranges grow densely, and fruits are thus easily occluded by one another or by the foliage. The occluded state of the oranges may vary when fruit images are acquired from different viewpoints. In this case, the abovementioned video-based counting methods may exhibit a tracking target loss owing to the complex occlusion present in global video sequences.

In this paper, using oranges as the target fruit, a deep-learning-based in-field orange fruit counting method is proposed, which includes the fruit detection algorithm OrangeYolo and the fruit tracking algorithm OrangeSort. OrangeYolo was modified from the object detection algorithm Yolov3 [24]. The network architecture was adapted to enable small-scale target detection by matching the feature map receptive field [25] with the target scale. A dual-attention multiscale fusion module was also introduced based on channel attention and spatial attention. OrangeSort is modified from the object tracking algorithm Sort [28]. A motion displacement estimation tracking algorithm was established, and a specific tracking region counting strategy was formulated to avoid the double-counting problem caused by the complex occlusion of fruits in global video sequences.

## Method

### Overall structure of the proposed algorithm

This study uses the same dataset acquisition method and criteria that were adopted in our previous work [38]. A field rover equipped with the DJI Osmo Action camera (DJI Technology Co., Ltd., ShenZhen, China) was used to acquire the video by moving at a uniform speed (2 m/s) along the tree rows.

**DJI Osmo Action camera:** The camera shooting direction was perpendicular to the tree rows. The camera has a frame rate of 60 fps, a resolution of 1920 × 1080, a 1/2.3 CMOS sensor, and a field of vision (FOV) of 145° F2.8.

**Our self-developed field rover:** For better operation in the orchard, we have customized a field rover. It has a length, width, and height of 120 × 90 × 70 cm, is motor driven through an ROS control interface, and has an automatic cruise function, which allows it to travel through the orchard according to a set track and speed. The functions are close to those of existing orchard work assistance robots already in agricultural use.

**Figure 1:**
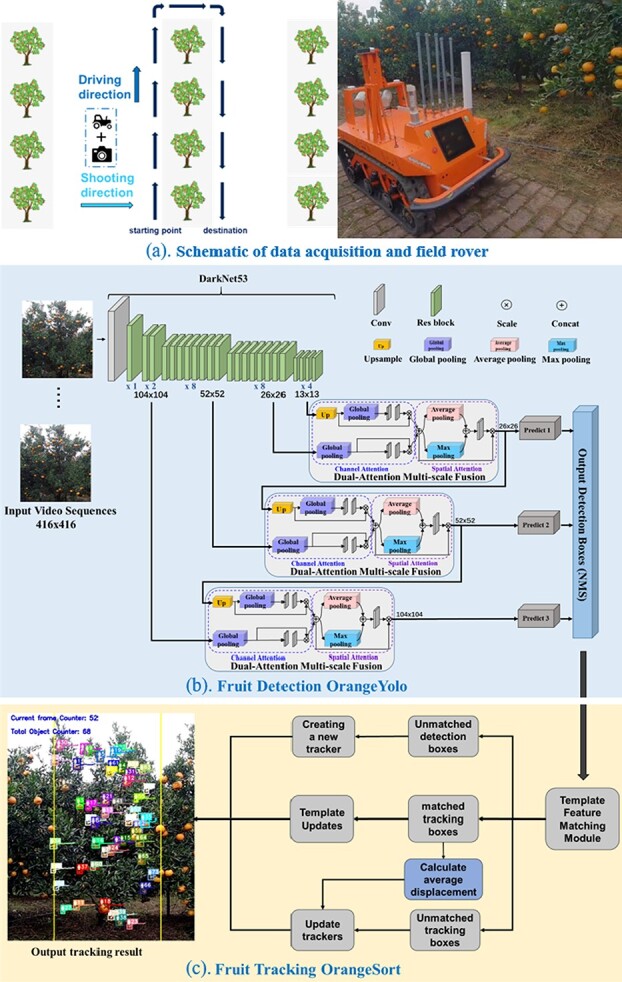
Overall structure of the algorithm. (**a**) Schematic of the acquisition method and rover. (**b**) Input video sequence (left) and detail of OrangeYolo (right). (**c**) Detail of OrangeSort (right corner) and the tracking result (left).

Figure 1 shows a schematic of the data acquisition method and field rover, as well as the overall structure of the fruit detection and tracking counting algorithm, which consists of the OrangeYolo and OrangeSort modules. The input is the video sequence from the orchard field. First, OrangeYolo detects the fruit frame-by-frame. The information for each detected fruit is fed to OrangeSort, which assigns a unique tracking ID to each individual fruit and correlates the same fruit data in different video consequences. Finally, the number of tracking IDs is counted.

**Figure 2:**
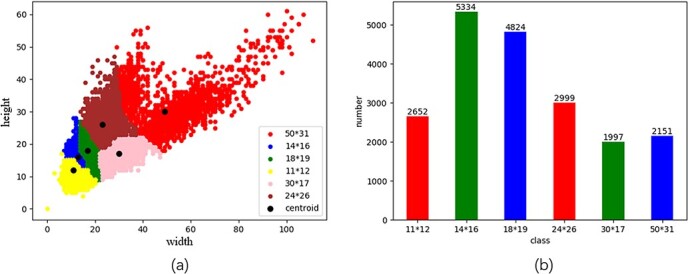
Results of k-means clustering analysis. (**a**) The k-means clustering yields results of 11 × 12, 14 × 16, 18 × 19, 24 × 16, 30 × 17, and 50 × 31. Each point on the scatter plot represents a labeled fruit target, and the horizontal and vertical coordinates indicate the width and height of the target, respectively. (**b**) The specific number distribution of the k-means clustering results.

**Figure 3:**
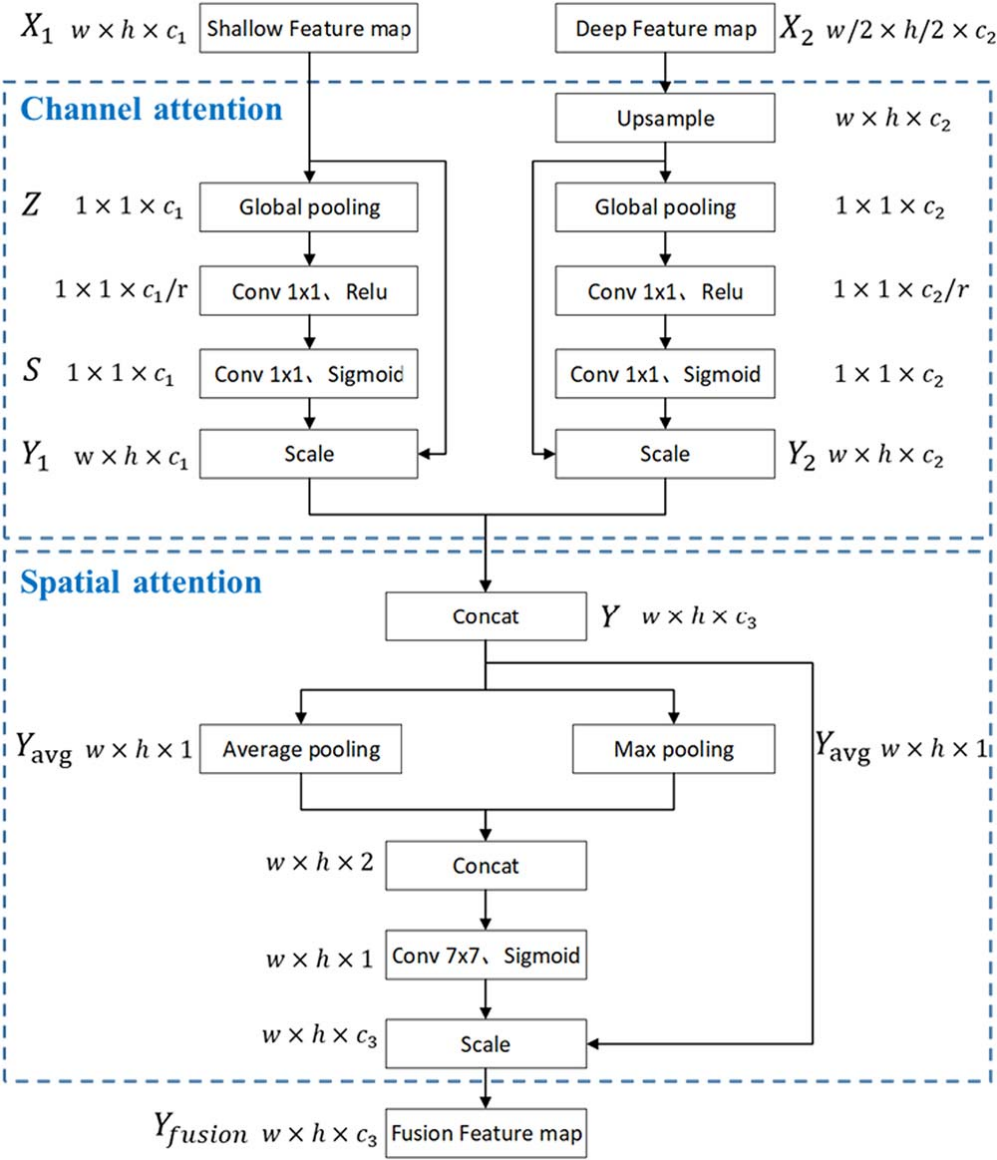
Dual-attention multiscale fusion module.

### OrangeYolo

The network structure of the OrangeYolo detection algorithm is shown in Figure 1(a). First, DarkNet53 [24] was used as the algorithm backbone network to extract the target features. DarkNet53 has been used by many researchers as a backbone network owing to its simplicity, efficiency [30, 31], and effectiveness in enhancing detection performance without modifying network structure and incurring additional computational costs. Second, in order to be able to detect targets at various scales in the images, this study analyzes the target scale characteristics of the field fruit dataset. Based on the receptive field scale matching principle, three detection branches were designed in the 18th, 26th, and 43rd convolutional layers of the backbone network to detect targets of various scales in the images. The three detection branches involve output feature maps with resolutions of 104 × 104, 52 × 52, and 26 × 26 to detect small-, medium-, and large-scale targets, respectively. Finally, to effectively fuse the features among the different convolutional layers, a dual-attention multiscale fusion module based on channel attention and spatial attention has been adopted. This network can fuse the semantic features of the deep network with the shallow texture detail features to enrich the semantic features of the shallow network and enhance the target detection accuracy. The fruit detection boxes of the three detection branches are fed to the non-maximal suppression algorithm to filter out low-scoring and overlapping boxes. The final retained box is the fruit detection result.

**Figure 4:**
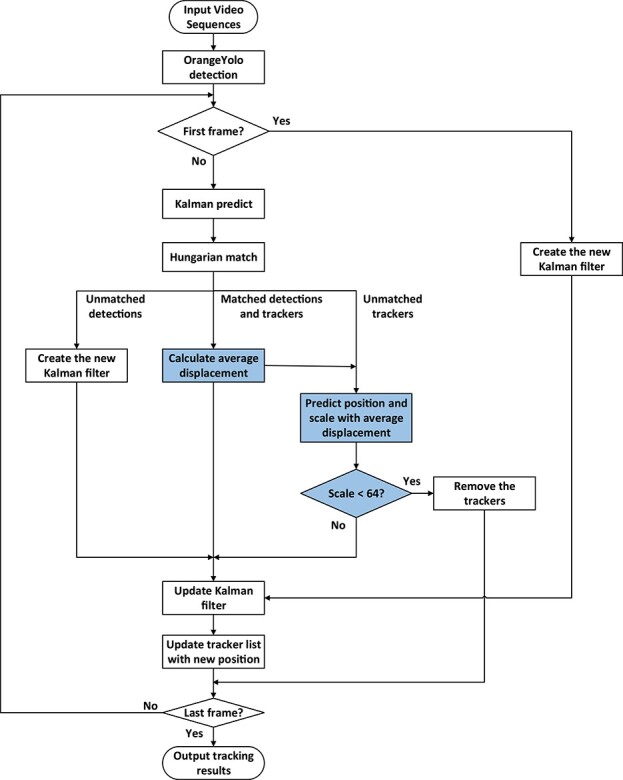
OrangeSort tracking algorithm flow.

### Receptive field scale analysis

Luo et al. [25] examined the receptive field problem in deep convolutional networks and introduced the concept of the effective receptive field. They noted that the best detection results are obtained only when the effective receptive field scale matches the target scale. On the left side of Figure 7 (Appendix Figure), the blue and red boxes indicate the receptive field of the feature map and the effective receptive field area, respectively. According to the abovementioned principle, the effective receptive field area must cover the complete target, as shown on the right side of Figure 7 (Appendix Figure). The receptive field scale can be defined as:

(1) \begin{equation*} {R}_k=1+\sum_{j=1}^k\left({F}_j-1\right)\prod_{i=0}^{j-1}{S}_i \end{equation*}

Here, ${R}_k$ denotes the receptive field size of the *k*-th convolutional layer, ${F}_j$ denotes the convolutional kernel size of the *j*-th convolutional layer, and ${S}_i$ denotes the step of the *i*-th convolutional layer.

The shallow and deep network feature maps have a small and large receptive field size and can detect small-scale and large-scale targets, respectively. To determine the network layers that are more suitable for detecting the field fruit targets, the collected field fruit dataset was annotated, and histogram statistical analysis was performed to explore the characteristics of the field fruit images.

To analyze the characteristics of the fruit scale size distribution, the k-means clustering statistics of the experimental dataset were derived [27]. As shown in Figure 2, k-means clustering produced results of 11 × 12, 14 × 16, 18 × 19, 24 × 16, 30 × 17, and 50 × 31. Each point in the scatter plot in Figure 2(a) represents a labeled fruit target, and the horizontal and vertical coordinates indicate the width and height of the target. Figure 2(b) shows the specific number distribution of the k-means clustering results. Based on all definition criteria pertaining to small targets, such as a target size less than 32 × 32, as defined by the coco dataset [32], or a target with a scale less than one-tenth that of the original image, most of the fruit targets in the field fruit image dataset can be considered small targets. The results of the receptive field scale and k-means clustering analysis of the fruit dataset from the DarkNet53 network and those obtained through the principle of receptive field scale matching (Equation 1) indicate that:

1) Conv9 feature maps with a receptive field scale of 29 × 29 are the most suitable for detecting fruit targets at scales of 11 × 12, 14 × 16, and 18 × 19.

2) Conv26 feature maps with a receptive field scale of 165 × 165 are the most suitable for detecting fruit targets at scales of 24 × 16, 30 × 17, and 50 × 31.

3) Because certain large-scale targets remain in the image, Conv43 was chosen as the third detection branch and retained the size of the anchor boxes designed by Yolov3 in this layer.

4) The receptive field scale of Conv53 feature maps is not applicable to the in-field fruit detection task. To better utilize these features in the corresponding network layer, they were fused with the shallow network for feature fusion to enrich the semantic features of the shallow network and enhance small-scale fruit detection accuracy.

**Table 1:** Analysis of fruit occlusion status in global video sequences.

**Fruit occlusion status**	**Fruit position and occlusion status**			**Effectivecounting area**
	**Occlusion of initial area (Y/N)**	**Occlusion of intermediate area (Y/N)**	**Occlusion of leaving area (Y/N)**	
A	N	N	N	Intermediate area
B	**Y**	N	N	
C	**Y**	N	**Y**	
D	N	N	**Y**	
E	N	**Y**	N	Initial and leaving area
F	N	**Y**	**Y**	Initial area
G	**Y**	**Y**	N	Leaving area

**Table 2:** Orange detection results. Rows 1–4 correspond to the training and testing data for 1025 and 440 images, respectively. The last row of the table indicates the test results for 182 images taken in a standard orchard.

**Model**	**Precision**	**Recall**	**F1-score**	**AP**	**FPS**	**Ground truth**
**Yolov3**	0.88	0.86	0.873	0.905	83.3	19466
**Yolov4**	0.862	0.877	0.854	0.911	71.4	19466
**Yolov5**	0.892	0.874	0.883	0.917	58.9	19466
**OrangeYolo (proposed)**	**0.902**	**0.893**	**0.897**	**0.938**	**83.3**	**19466**
OrangeYolo (proposed)^*^	**0.913**	**0.919**	**0.916**	**0.957**	**83.3**	**4441**

### Dual-attention multiscale fusion module

The receptive field scales and feature information of the feature maps from different convolutional layers are considerably different. For example, the shallow network features have small receptive field scales and focus on the color and textural details of the image, whereas the deep network features have larger receptive field scales and contain abundant semantic features. To exploit the features of different convolutional layers to accomplish vision tasks with high performance, several multiscale fusion strategies have been proposed [33–35]. These fusion strategies, however, generally only up-sample the feature maps of different sizes to ensure that the two feature maps are of the same size and directly implement dimensional concatenation.

Although the fused features are derived from different deep convolutional layers, the algorithms do not consider the correlation properties between the different feature maps and thus cannot fully fuse and utilize the effective information from different feature maps. In recent years, the attention mechanism of deep learning has received considerable attention for the enhancement of feature quality. Hu et al. [36] proposed SE-Net to establish the relationship between feature channels, obtain the importance of each channel via the adaptive learning method, and assign different weights according to channel importance to enhance their effectiveness. In contrast to the SE-Net attention module, which focuses only on the weight relationship of each feature channel, the CBAM [37] attention module integrates the spatial features and weight relationship of each channel and can achieve excellent results on other image recognition tasks.

Oranges tend to grow in clusters, and the spatial characteristics of image features must therefore be considered to enhance the discriminative ability of the local information and accurately detect each fruit target. Based on the characteristics of field fruit data, a multiscale fusion module was established based on channel attention and spatial attention mechanisms. First, the channel attention module is separately applied to two feature maps from different convolutional layers, thereby realizing adaptive learning based on the importance of each channel. Next, after channel enhancement, the two feature maps are dimensionally connected and input to the spatial attention module to enhance the response level to each target region. The structure of this module that contains channel and spatial attention, that is, the dual-attention multiscale fusion module, is shown in Figure 3.

**Figure 5:**
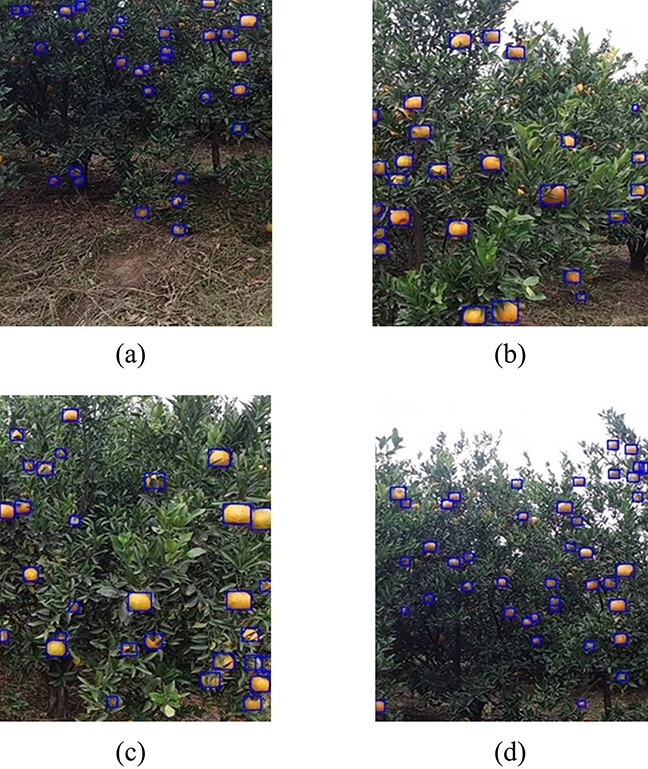
Visualization of detection results (blue bounding box). (**a**), (**b**), and (**c**) indicate the visualization results for detection of small-scale targets, medium-scale targets, and large-scale targets, respectively, and (**d**) indicates the detection results for the standard dataset. Note that orange fruit targets of different scales in the image can be detected accurately.

**Table 3:** Ablation experiment results for four components. RF (Receptive field) matched: sensory field scale matching and optimized anchor box strategy; Mosaic augmentation: mosaic data augmentation method; Channel attention: channel attention enhancement module; and Dual-attention: dual-attention mechanism fusion module.

**Method**	**RF matched**	**Mosaic augmentation**	**Channel attention**	**Dual attention**	**F1-score**	**AP**
					0.873	0.905
	**√**				0.887	0.928
OrangeYolo	**√**	**√**			0.89	0.93
	**√**	**√**	**√**		0.892	0.933
	**√**	**√**		**√**	**0.897**	**0.938**

**Channel attention:** The input includes two features from different depth network layers with different dimensions. Shallow feature ${X}_1$ and deep feature ${X}_2$ have dimensions $W\times H\times {c}_1$ and $\frac{W}{2}\times \frac{H}{2}\times {c}_2$, respectively. First, the deep feature ${X}_2$ is up-sampled by a factor of 2 to ensure that it has the same scale as the shallow feature${X}_1$. Next, the channel attention operation is performed on the two features separately. Let us consider feature ${X}_1$ as an example. First, the global pooling layer compresses each two-dimensional channel of feature ${X}_1$ to a real number. Each real number characterizes the global distribution of the responses over the feature channels, as shown in Equation 2, where ${z}_c$ denotes the response value of channel *c* in feature${X}_1$, ${x}_c$ represents the *c*-th channel of feature ${X}_1$, and *H* and *W* denote the height and width of feature *U*, respectively.

After developing the two 1 × 1 convolutional layers to establish the correlation between the channels, the sigmoid activation layer can obtain *c* weights normalized between 0 and 1. In Equation 3, *σ* and *δ* denote the sigmoid activation function and the ReLU activation function, respectively, ${W}_1$ and ${W}_2$ denote the weight parameters of the first and second convolutional layers, respectively, and *S* is the normalized weight matrix. The scale operation assigns the normalized weights to each channel of feature ${X}_1$ to obtain a channel-weighted feature map ${Y}_1$ with dimensions *w* × *h*×${c}_2$. The scale is calculated as shown in Equation 4. ${y}_c$ denotes the feature vector of the *c*-th channel in the feature map ${Y}_1$. Finally, the channel attention-enhanced features ${Y}_1$ and ${Y}_2$ are dimensionally concatenated to obtain the fused channel-attention-enhanced features *Y*.

(2) \begin{equation*} {z}_c=\frac{1}{H\times W}\sum_{i=1}^H\sum_{j=1}^W{x}_c\left(i,j\right) \end{equation*}

(3) \begin{equation*} S={F}_s\left(Z,{W}_1,{W}_2\right)=\sigma \left({W}_2\bullet \delta \left({W}_1Z\right)\right) \end{equation*}

(4) \begin{equation*} {y}_c={F}_{Scale}\left({x}_c,{s}_c\right)={s}_c{x}_c \end{equation*}

**Spatial attention:** First, the average pooling and maximum pooling operations are performed on *Y* to calculate the spatial dimensional weights. Next, the spatial weights are weighted with *Y* to obtain the channel and spatial attention enhancement features ${Y}_{fusion}$. In Equation 5, ${Y}_{avg}$ and ${Y}_{max}$ represent the average pooled features and maximum pooled features generated by the average pooling and maximum pooling operations on *Y*, respectively. ${Y}_{concat}$ represents the feature after the dimensional concatenation of ${Y}_{avg}$ and ${Y}_{max}$. *σ* represents the sigmoid operation, ${f}^{7\ast 7}$ represents the convolution operation with a convolution kernel size of 7 × 7, and ${F}_{Scale}$ represents the dimensional concatenation operation.

(5) \begin{align*}{Y}_{fusion}&={F}_{Scale}\left(\sigma \left({f}^{7\ast 7}\left(\left[ AvgPool(Y); MaxPool(Y)\right]\right)\right),Y\right) \notag\\&={F}_{Scale}\left(\sigma \left({f}^{7\ast 7}\left(\left[{Y}_{avg},{Y}_{max}\right]\right)\right),Y\right)\notag\\ &={F}_{Scale}\left(\sigma \left({f}^{7\ast 7}\left({Y}_{concat}^S\right)\right),Y\right) \end{align*}

### Data augmentation

In the previously presented analysis of small-scale target characteristics, small targets were noted to be challenging to detect. Recently, some researchers have examined techniques for performing image augmentation for small targets in the data pre-processing stage to enhance the recognition ability of detectors for small targets [40, 41]. The Mosaic data augmentation method can combine multiple training images into one image for training according to the set mini-batch, thereby diversifying the target scale and enriching the image context. Figure 8 (Appendix Figure) shows the results of the mosaic data augmentation.

### OrangeSort

In this study, we fully analyze the fruit distribution characteristics in the orchard scene and propose the OrangeSort algorithm for citrus fruit tracking. First, we improve the algorithm based on the mainstream multi-target tracking algorithm “sort” and proposed a motion displacement estimation algorithm that can significantly reduce the errors caused by the double-counting problem. Second, we propose a region counting strategy that can solve the problem of counting errors caused by complex occlusion problems in orchard scenes. 

**Figure 6:**
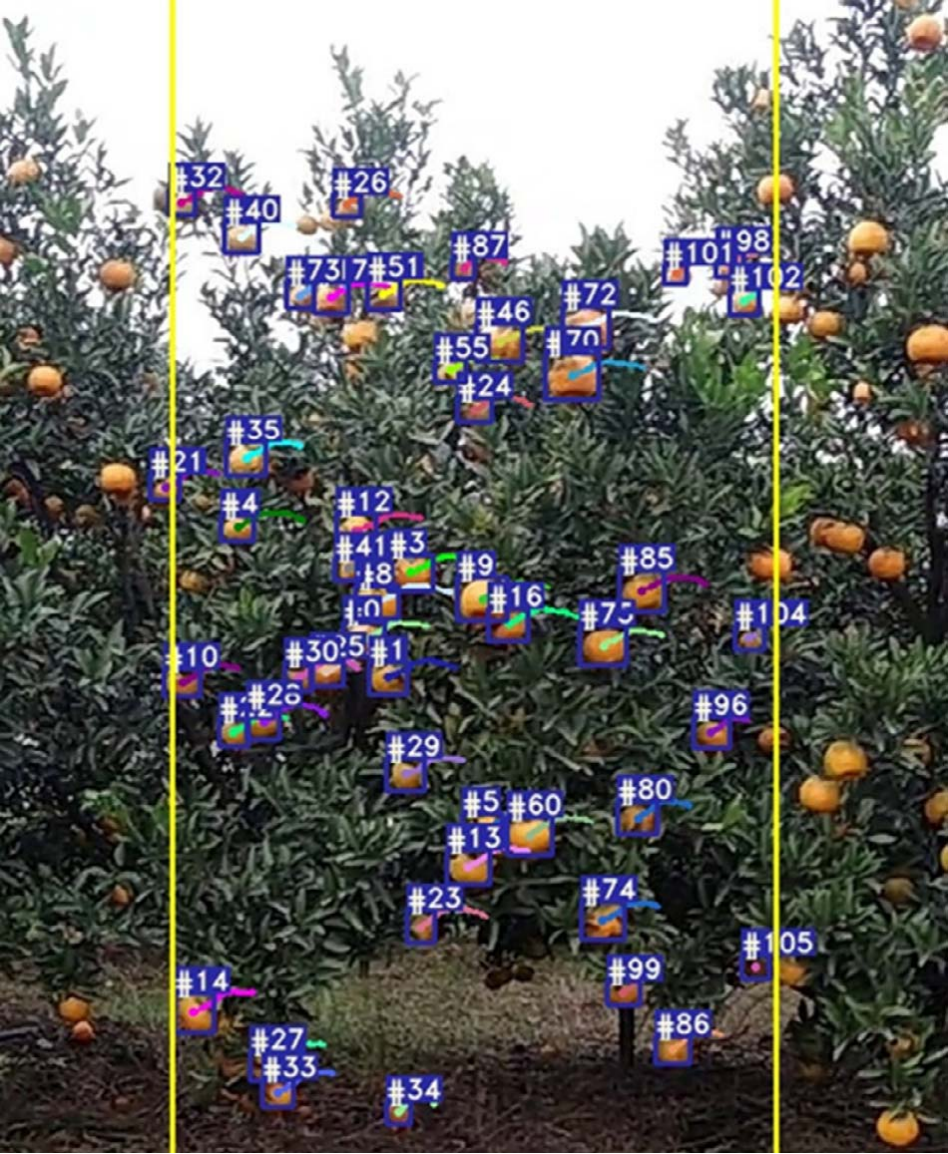
OrangeSort tracking count example: blue boxes indicate each fruit that was target tracked, and the number represents the ID assigned to each fruit target. The area between the two yellow lines is the tracking counting region used for OrangeSort^*^. Video available at: https://youtu.be/2BrPI2Vw_TM.

### Motion displacement estimation

In real orchards, fruit grows densely and overlaps heavily. This is a significant problem for tracking algorithms in computer vision. Although the mainstream sort algorithm [28] performs satisfactorily in many areas of application, double-counting occurs in practice. This problem can have a significant impact on the performance of the algorithm in fruit counting. When a tracking target is occluded or cannot be detected in several consecutive frames, the target tracker ID is written off. When the target can be accurately detected again in subsequent frames, however, a new tracker ID is created for the target because a tracker no longer exists for this target.

In a field fruit video sequence, all the objects are stationary, and the trajectory of each fruit is approximately the same as the distance the camera moves during a video sequence. Therefore, when the algorithm is unable to track a particular target correctly, it can consider using the average motion displacement over the correctly tracked target to estimate the position of that target in the current frame.

The motion displacement estimation tracking method involves two steps: 1) Tracking target center position estimation and 2) Tracking target scale estimation.

**Table 4:** Counting results of OrangeSort, Sort [28], and DeepSort [29] tracking algorithms.

**Video sequence ID**	**Counting method**	**Manual counting**	**Number of fruit counts**	**Number of correct counts**	**Number of false counts**	**Number of double-counting instances**	**Counting error**
1	Sort	165	277	147	54	76	0.6788
	Deepsort		252	142	14	96	0.5273
	OrangeSort		189	145	22	22	0.1455
	**OrangeSort** ^*^		166	132	27	7	**0.0061**
2	Sort		72	40	26	6	0.125
	Deepsort	64	105	39	33	33	0.6406
	OrangeSort		70	40	25	5	0.0938
	**OrangeSort** ^*^		57	40	13	4	**0.1094**
3	Sort		113	66	26	21	0.6143
	Deepsort	70	212	65	49	98	2.0286
	OrangeSort		96	66	16	14	0.3714
	**OrangeSort** ^*^		87	61	14	12	**0.2429**
	Sort		270	114	111	45	0.4754
4	Deepsort	183	515	115	82	318	1.8142
	OrangeSort		211	114	66	31	0.1265
	**OrangeSort** ^*^		170	93	58	19	**0.071**
5	Sort		117	66	33	18	0.2717
	Deepsort	92	174	64	31	79	0.8913
	OrangeSort		106	65	27	14	0.1522
	**OrangeSort** ^*^		95	65	19	11	**0.0326**
6	Sort		381	195	152	34	0.5363
	Deepsort	248	588	202	104	282	1.371
	OrangeSort		327	190	115	22	0.3185
	**OrangeSort** ^*^		242	170	59	13	**0.0242**

Note: OrangeSort^*^ represents the counting results of the OrangeSort algorithm using the specific counting region strategy.

**(1) Tracking target center position estimation:** The displacement of the correct tracking target center point in the previous frame is used to estimate the corresponding center point in the current frame image. The coordinates of the center point of the tracked target are calculated using Equation 6. ${x}_i$ and ${x}_i^{\prime }$ denote the x-axis coordinate of the center point of the *i*-th correctly tracked target in the current frame and previous frame, respectively, *n* denotes the number of correctly tracked targets in the current frame, and $\Delta x$ and $\Delta y$ denote the average displacement in the x- and y-axis directions of the current and previous frames of the *n* correctly tracked targets. The position (${x}^{\prime },{y}^{\prime }$) of the center point ($x,y$) of the unmatched tracking target in the current frame can be calculated using ∆*x* and ∆*y*.

(6) \begin{align*} \left({x}^{\prime },{y}^{\prime}\right)&=\left(x+\Delta x,y+\Delta y\right)\notag\\&=\left(x+\frac{\sum_{i=1}^n{x}_i-{x}_i^{\prime }}{n},y+\frac{\sum_{i=1}^n{y}_i-{y}_i^{\prime }}{n}\ \right) \end{align*}

The accuracy of multi-target tracking algorithms based on frame-by-frame detection is considerably affected by the detection results, which can easily lead to the loss of tracked targets in the following two cases: 1) the target is occluded and cannot be detected by the detector; 2) the target appears clearly in the video but is missed by the detector in certain frames.

**Table 5:** Counting standard deviations of OrangeSort, Sort [28], and DeepSort [29] tracking algorithms.

**Counting method**	**Sort**	**DeepSort**	**OrangeSort**	OrangeSort^*^
Standard deviation	0.4741	1.3975	0.2555	0.08

If the detector cannot provide the location of the target to the tracking algorithm, the corresponding tracking target in the current frame is identified as an unmatched tracking target. Compared to the case in which the tracking target appears clearly in the video but is missed by the detector, the tracking target is more likely to be lost due to occlusion. In general, the occlusion of the tracking target in the video is a gradual process from slight to heavy occlusion. At this time, the pixel area of the blocked fruit in the image will gradually decrease, as shown in Figure 9 (Appendix Figure). Therefore, the following tracking target scale estimation method was established to manage the situation in which the tracking target is lost owing to the occlusion condition.

**(2) Tracking target scale estimation:** After determination of the center position of the tracking target, the width and height dimensions of the tracking target are calculated to obtain the tracking target position area in the current frame. The width and height of the tracking target in the current frame image are estimated. After determination of the location of the center point of the tracking target, the scale size of the target in the previous frame is considered as the base. The scale decay factor *α* is used to estimate the scale size of the target in the current frame, as shown in Equation 7, where ${w}_1\ \mathrm{and}\ {h}_1$ denote the width and height of the tracking target in the current frame, and $w$ and $h$ denote the width and height of the tracking target in the previous frame, respectively.

(7) \begin{equation*} \left({w}_1,{h}_1\right)=\left(w\ast \alpha, h\ast \alpha \right) \end{equation*}

Because the occlusion of the target in the video sequence is a gradual change, the implementation of the motion displacement estimation operation on an unmatched tracking target may cause the estimated tracking target to exhibit a scale decay. The motion displacement estimation tracking strategy is formulated as follows: 1) If the scale of the tracking target decays continuously after a certain level, the target is considered to not appear in the video owing to complete occlusion, and the tracking target is removed from the tracking list. 2) If the target is not matched by the tracked target owing to slight occlusion or misdetection, OrangeYolo can accurately detect the target again in several consecutive frames, thereby providing data support for the tracking algorithm to enable the tracker to track the target again.

Based on the analysis of the motion characteristics of the target in the field fruit video sequence, the steps involved in OrangeSort can be enumerated as follows, as illustrated in Figure 4, where the blue boxed portion is the improvement point concerning the motion displacement estimation.

1) First, the proposed detection algorithm OrangeYolo is used to detect the fruit in each frame of the image. Then a detection box is generated for each fruit target and input to the tracking algorithm OrangeSort.

2) The proposed tracking algorithm OrangeSort determines whether the current image is in the first frame. If it is, a new ID is assigned to each fruit detection box, which is the tracking result of the current frame. If it is not, the detection result of the current frame is matched with the tracking result using the Hungarian algorithm. The boxes are divided into three categories, “unmatched detection box”, “correctly matched detection box”, and “unmatched tracking box”.

3) The three categories are processed as follows.

In the **unmatched detection box case**, a particular detection result for the current frame does not match any of the tracking results from the previous frame. This indicates that the target is appearing in the image for the first time or is being detected for the first time; therefore, a new tracker ID is created directly.

In the **correctly matched detection box** case, the detection result is correctly associated with a tracking target. First, the average displacement of all correctly matched detection frames in the coordinates of the current frame and the previous frame is calculated, and the state and tracking list of the corresponding Kalman trackers is updated using the tracking results of the current frame.

In the **unmatched tracking box** case, a particular tracking result from the previous frame cannot be matched with any of the detected results from the current frame. At this point, the algorithm steps are as follows. First, the coordinates of the center point of the unmatched tracking box in the current frame are calculated, considering the average displacement of all correctly matched detection frames. Then, the width and height of the tracking target in the current frame are calculated based on the target scale of the previous frame and from *α*. If the scale is smaller than the threshold, the tracking target is considered to be possibly heavily occluded and not present in the image for a long time, and the tracker is therefore deleted.

4) The algorithm determines if the result pertains to the last frame of the video. If not, it loops through steps 2 and 3. If so, it outputs all tracking results.

### Tracking strategy determination

To alleviate the problems of existing video-based methods, which cannot address complex occlusion situations that may exist in global video sequences, an effective region tracking counting strategy is established by analyzing the characteristics of video sequences of fruit in the field.

Frequent cases of complex and variable fruit occlusion in the video sequences have a considerable influence on tracking performance and are one of the key factors that lead to double counting, as shown in Figure 10 (Appendix Figure) and Figure 11 (Appendix Figure).

**Fruit position classification:** Fruit position states were classified into three categories according to their different positions in the image, as shown in Figure 11 (Appendix Figure): initial position, intermediate position, and leaving position.

Initial position: In frame 29, the fruit indicated by the green arrow is only slightly in the field of view of the camera.

Intermediate position: In frame 71, the fruit indicated by the green arrow is approximately in the central area of the image.

Leaving position: In frame 100, the fruit indicated by the green arrow is about to leave the field of view.

**Counting region determination:** Based on the fact that oranges may have different occlusion states at each position, all possible complex occlusion cases of fruits in the global video sequence were analyzed. Table 1 lists all possible occlusion conditions of the fruit in the global video sequence from entering to leaving the camera view. According to the statistical analysis of fruit occlusion status for the global video sequences of certain trees, conditions A, B, C, and D account for the largest proportion of all global video sequence occlusion conditions. Therefore, the intermediate area of the video was considered as the core tracking counting region. Conditions E, F, and G were considered to maximize the advantages of the video-sequence-based tracking counting method and to reduce the influence of complex occlusion in the global video sequence.

In “Fruit occlusion status”, Y indicates that there is occlusion in this region, and N indicates that there is no occlusion in this region. The final tracking counting region contains part of the initial area and leaving area. In cases A, B, C, and D, the intermediate positions do not exhibit any occlusion. Consequently, it is only necessary to count the fruits in this position, thus reducing the computational resources required. In conditions E, F, and G, the fruits are occluded in the intermediate position and not occluded in either the initial or leaving position. To count the fruits, the counting area must include part of the initial position area and leaving position area.

The width of the tracking count area is 3/5^th^ of the original video sequence width. The regions 1/5^th^ after the initial position and 1/5^th^ prior to the leaving position are invalid regions. Experimental analysis indicates that the set specific region counting strategy can effectively overcome the influence of occlusion on tracking counting, with reduced tracking computation and enhanced operation speed.

**Figure 7:**
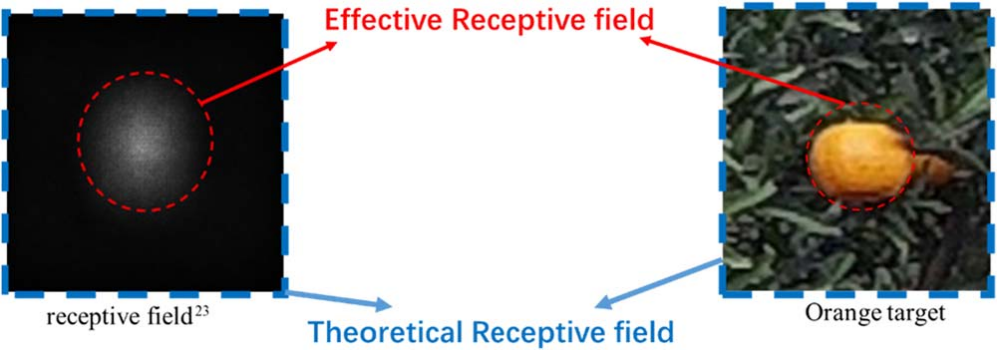
Feature map receptive field matched to the target. Left, the blue and red boxes indicate the receptive field of the feature map and the effective receptive field area, respectively. Right, the effective receptive field area for fruit detection.

**Figure 8:**
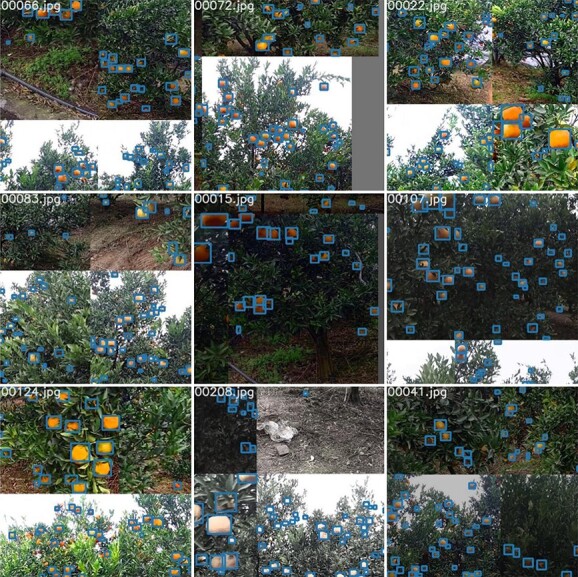
Mosaic augmentation results.

**Figure 9:**
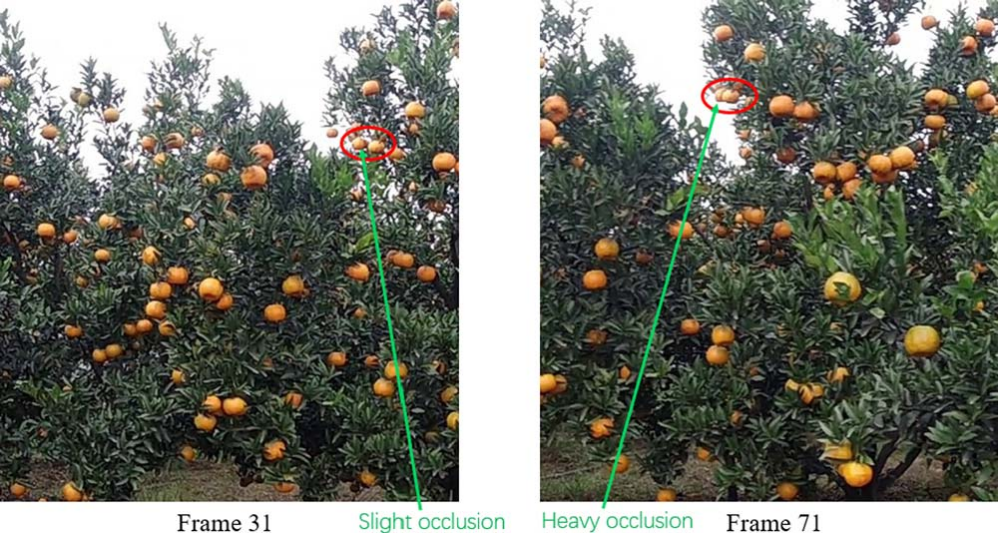
Change in shading of the same fruit in the video sequence. The green arrow shows fruit A in frame 31 almost side-by-side with another fruit, fruit B. In frame 71, most of fruit A has been obscured by fruit B. The pixel area of fruit A is gradually decreasing.

## Experiment

### Data acquisition

The data acquisition is performed in two orange orchards in Meishan city, Sichuan Province, China, (29°55′25′′N 104°17′23″E, 30°14′26′′N 104°06′04″E), and the video was acquired using a field rover equipped with a DJI Osmo Action camera.

**Figure 10:**
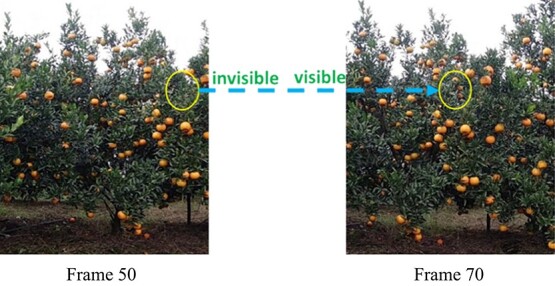
Visible state of the fruit. In frame 50, the marked yellow area enters the camera view, and the fruit in the area is completely occluded by leaves. However, with a change in acquisition perspective, the fruit in the yellow area in frame 70 can be clearly observed.

The rationality of the data set collection method can be referred to our previous research work [39]. The field rover moves at an even speed along the direction of the tree rows. The camera is perpendicular to the tree row direction when acquiring the video. To observe a sufficient number of fruits, the full view of each fruit tree must be captured, and the vertical direction of each frame must cover the top and bottom of the fruit tree, as shown in Figure 10 (Appendix Figure).

To ensure a diversity of data when selecting the image dataset for the fruit detection algorithm, data acquisition was randomly performed at different locations in the orchard. A random cropping strategy was also implemented on the original video sequences to produce 1465 samples of image datasets with different target scales and different shooting angles for training and testing the target detection algorithm. Among these, the test set of 182 images is the standard dataset. During acquisition, the shooting distance was kept consistent, and the shooting angle was kept constant, ensuring that the vertical height just encompassed the top of the tree canopy and the bottom of the tree trunk.

**Figure 11:**
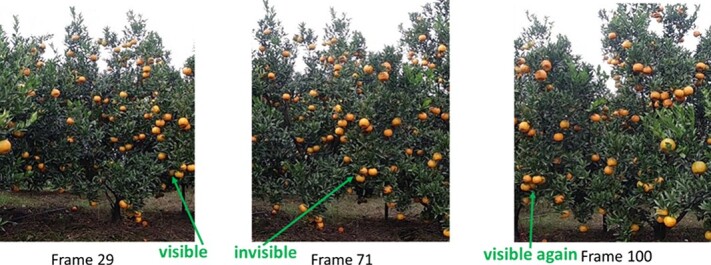
Video sequence occlusion analysis. The orange indicated by the green arrow can be clearly observed in frame 29; however, as the acquisition device moves, the orange cannot be observed in frame 71 because it has been occluded by an orange next to it. Furthermore, immediately before the fruit leaves the field of view of the camera, the fruit indicated by the green arrow appears clearly in frame 100, owing to a change in acquisition viewpoint.

**Figure 12:**
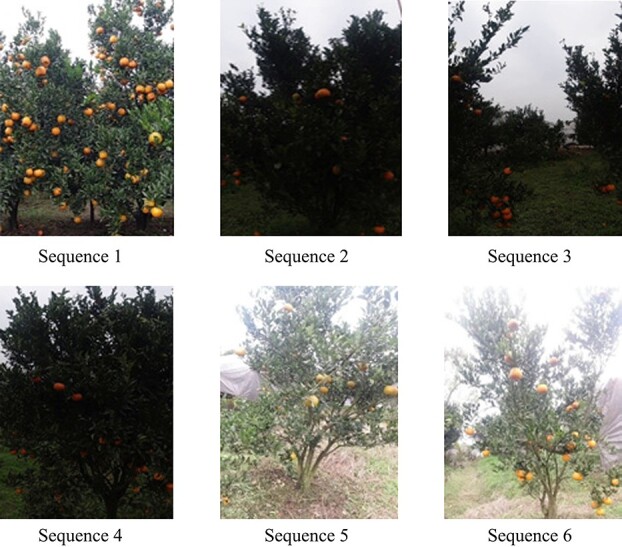
Video sequence display. The video sequences contain a variety of complex occlusions, scale variations, and different shooting conditions that are frequently encountered in practical application. Video sequence 1 involves dense fruit growth, video sequences 2–4 are poorly lit, and video sequences 5–6 are overexposed, with bumps and dramatic camera shake during shooting.

### Experimental details

A computer with a GeForce GTX 1080 Ti GPU and an Intel i7 8th generation CPU was used to train and test OrangeYolo. Specifically, 1465 data samples were randomly divided into training and testing sets in a ratio of 7:3 (for more details, please refer to our published dataset: https://github.com/I3-Laboratory/orange-dataset). The SGD optimizer was used to optimize the gradient, with a momentum, weight decay, and initial learning rate of 0.9, 0.0005, and 0.01, respectively.

Five evaluation metrics were used to verify the effectiveness of OrangeYolo: precision, recall, F1-score, average precision (AP), and detection speed (FPS). The detection results were divided into true positives (TPs) and false positives (FPs). TP indicates that a manually marked orange target is correctly detected, and FP indicates that a non-target is incorrectly detected as a target; for example, a leafy branch is mistakenly detected as an orange. Precision indicates the proportion of TPs in the detection results relative to TP + FP of all detection results, and it is calculated as shown in Equation 8. Recall indicates the proportion of TPs in the detection results relative to the number of manually marked samples, GroundTruth, and it can be calculated using Equation 9. The F1-score, calculated using Equation 10, takes into account both precision and recall to evaluate detection performance. A larger F1-score corresponds to higher detection performance. AP, which corresponds to the area under the precision and recall curves and the average of all precision values with recall between 0 and 1, indicates the overall performance of the detection model. AP can be calculated using Equation 11, where *p* and *r* indicate the precision and recall values, respectively.

(8) \begin{equation*} Precision=\frac{TP}{TP+ FP} \end{equation*}

(9) \begin{equation*} Recall=\frac{TP}{GroundTruth} \end{equation*}

(10) \begin{equation*} F1- score=\frac{2\ast Precision\ast Recall}{Precision+ Recall} \end{equation*}

(11) \begin{equation*} AP={\int}_0^1 Precision(r) dr \end{equation*}

To verify the effectiveness of OrangeSort, tracking counting experiments were performed using six video sequences containing 22 trees, and the results were compared with manual counting results for the video sequences. The accuracy of the count results is also expressed using the mean absolute error (MAE), which can be expressed by Equation 12.

(12) \begin{equation*} MAE=\frac{1}{m}\sum_{i=1}^m\left|\frac{y_{test}^{(i)}-{\hat{y}}_{test}^{(i)}}{{\hat{y}}_{test}^{(i)}}\right| \end{equation*}

where${y}_{test}^{(i)}$denotes the fruit counting result of the tracking algorithm in the *i*th video sequence,${\hat{y}}_{test}^{(i)}$ denotes the artificial fruit counting result in the *i*th video sequence, and *m* denotes the total number of video sequences. As shown in Figure 12 (Appendix Figure), the video sequences contain a variety of complex occlusions, scale variations, and different shooting conditions that are frequently encountered in practical applications.

### Comparative analysis of experimental results

**(1) Performance analysis of OrangeYolo:** Table 2 summarizes the various performance indicators of OrangeYolo. OrangeYolo outperforms the comparison algorithms [V3, V4] [24, 40] in terms of all five indicators, with an F1-score, AP, and FPS of 0.897, 0.938, and 83.3, respectively. The last row of Table 2 shows the test results on 182 images from the standard dataset. The proposed algorithm achieves an average accuracy of 0.957 on standard fruit images and an F1 value of 0.916. This demonstrates that the standard dataset obtained with a standardized shooting method can better help the detection algorithm to detect citrus fruits accurately. The inference time FPS is the same as that of Yolov3, and both are better than other comparison algorithms, demonstrating the practical application value of the proposed method. Next, ablation comparison experiments were performed to verify the effectiveness of the proposed receptive field scale matching strategy. As shown in Table 3, the detection performance could be effectively improved by using the receptive field matching strategy, and the F1-score and AP values were enhanced by 1.4% and 2.3%, respectively. The highest detection performance was achieved after using the dual-attention multiscale fusion mechanism, and the F1-score and AP values were enhanced by 0.7% and 0.8% from 0.89 and 0.93, respectively. Figure 5 shows the visualization results of the detection algorithm. It can be noted that orange fruit targets of different scales can be detected accurately in the image.

**2) Performance analysis of OrangeSort:** Figure 6 shows the OrangeSort results obtained using the specific counting region tracking strategy. Table 4 presents the counting results of the OrangeSort, Sort [28], and DeepSort [29] tracking algorithms tested on six video sequences. Six metrics are considered: number of fruit counts, number of correct counts, number of missed counts, number of false counts, number of double-counting instances, and counting error, where the counting error is the MAE of a single video sequence, which can be expressed as $MAE(m=1)$. The OrangeSort algorithm exhibits a significantly lower number of double-counting instances compared with the Sort and DeepSort algorithms. The total number of fruit counts is also closer to the number of manual counts, with counting errors of 0.1455, 0.0938, 0.3714, 0.1265, 0.1522, and 0.3185. OrangeSort^*^ indicates the counting results obtained using the specific counting region strategy. Although OrangeSort^*^ has slightly fewer correct counts, it effectively overcomes the ID switch problem in the tracking process and reduces the number of double-counted fruits. The counting results are close to the manual counting results, with counting errors of 0.0061, 0.1094, 0.2429, 0.071, 0.0326, and 0.0242. The data of video sequence 1 were collected in the orchard using standard lighting conditions, and the algorithm therefore gives the smallest counting error for this sequence. Video sequences 2–4 were collected in a poorly lit environment, and the overall counting error is larger. The light is worst in video sequence 3; it is almost impossible to distinguish the categories of fruits and leaves, and the counting error is therefore the largest. Video sequences 5–6 were collected in a strong light environment and were overexposed, with bumps and dramatic camera shakes. Most of the fruit targets could be distinguished, but there were still a few fruit targets that could not be tracked accurately, and the counting error was relatively high. In video sequence 2, OrangeSort counted 70 fruits, with 40 correct counts, 5 double-counting instances, and 25 error counts. OrangeSort^*^ counted 57 fruits, with 40 correct counts, 4 repeat counts, and 13 error counts. The counting errors of OrangeSort and OrangeSort^*^ were similar, with values of 0.0938 and 0.1094, respectively. However, OrangeSort counted more error samples by performing fruit tracking counts in the global region, resulting in nearly twice as many error counts as OrangeSort^*^. Consequently, the result obtained using OrangeSort^*^ was closer to the true value of the manual counts.

By computing the mean absolute error (MAE) of the six video sequences with the results in Table 5, we can conclude that OrangeSort^*^ produces the smallest counting error of 0.081 and a standard deviation of 0.8 compared with the Sort and OrangeSort algorithms (without implementing the counting region strategy). The counting results of OrangeSort^*^ are clearly closest to the manual counting results.

## Conclusion

This work improves the accuracy of fruit counting in terms of fruit detection and double-counting. Taking into account the small-scale characteristics of field orange data and the different occlusion statuses in the video sequence, a video-sequence-based fruit counting algorithm was established, including two sub-algorithms, OrangeYolo for fruit detection and OrangeSort for fruit tracking.

The network structure, designed based on the receptive field matching strategy and the dual attention fusion module, constitutes the fruit detection algorithm OrangeYolo, which achieves an AP value of 0.938 on the field orange detection dataset. A specific tracking region counting strategy and tracking algorithm based on motion displacement estimation constitute the OrangeSort tracking algorithm. Using six video sequences taken from two fields containing 22 trees as the validation dataset, the proposed method showed the best performance (MAE = 0.081, SD = 0.08) relative to video-based manual counting. These results demonstrate the practical value of the proposed method compared with other existing algorithms. Future work can be aimed at using 3D technology to locate fruit spatial coordinates to enable more accurate counting, and line tail turns will be explored further in subsequent research work. Furthermore, a lightweight network model on edge devices can be designed and deployed to accomplish lightweight real-time field fruit counting and to link the actual fruit number by considering the unseen part of the tree to achieve actual yield estimation.

## Author contributions

W.Z., J.W., Y. L. and W.G. conceived the ideas and designed methodology; H.L., Y. D., W.W., and Y. S. collected field data with self-designed field rover. All authors discussed, wrote the manuscript, and gave final approval for publication.

## Data availability

The dataset used during this study is available in a repository in accordance with funder data retention policies. We have published the dataset at GitHub (https://github.com/I3-Laboratory/orange-dataset).

## Conflict of interest statement

The authors declare that they have no conflicts of interest.

## Supplementary data

Supplementary data is available at *Horticulture Research* online.

## Supplementary Material

Web_Material_uhac003
