# Supplementary material for: Deep-learning-based in-field citrus fruit detection and tracking
Source: Hortic Res. 2022 Feb 11;9:uhac003. doi: 10.1093/hr/uhac003 (PMC9113225; doi:10.1093/hr/uhac003)
Supplement: Web_Material_uhac003 [file web_material_uhac003.zip › Appendix.docx]

**Appendix**


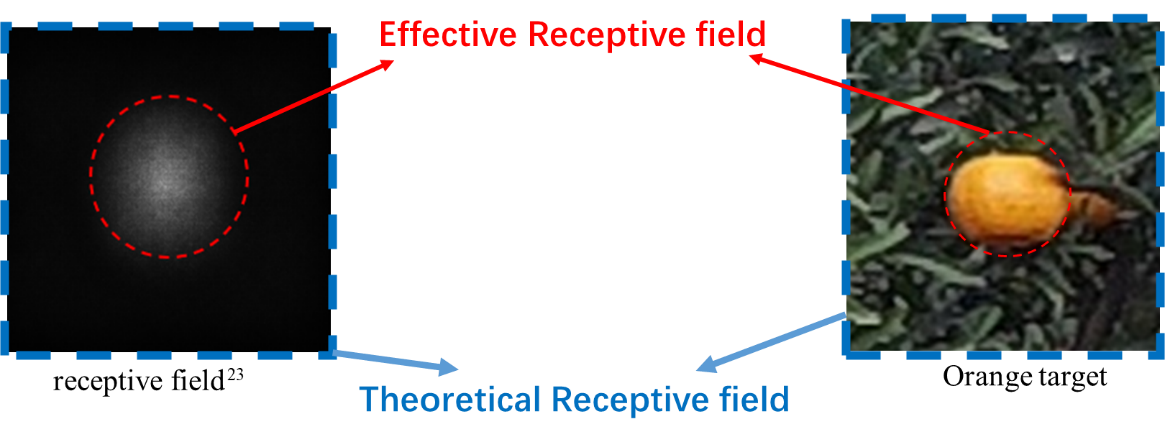


Appendix 1. Feature map receptive field matched to the target.

Left, the blue and red boxes indicate the receptive field of the feature map and effective receptive field area, respectively. Right, the effective receptive field area for fruit detection.


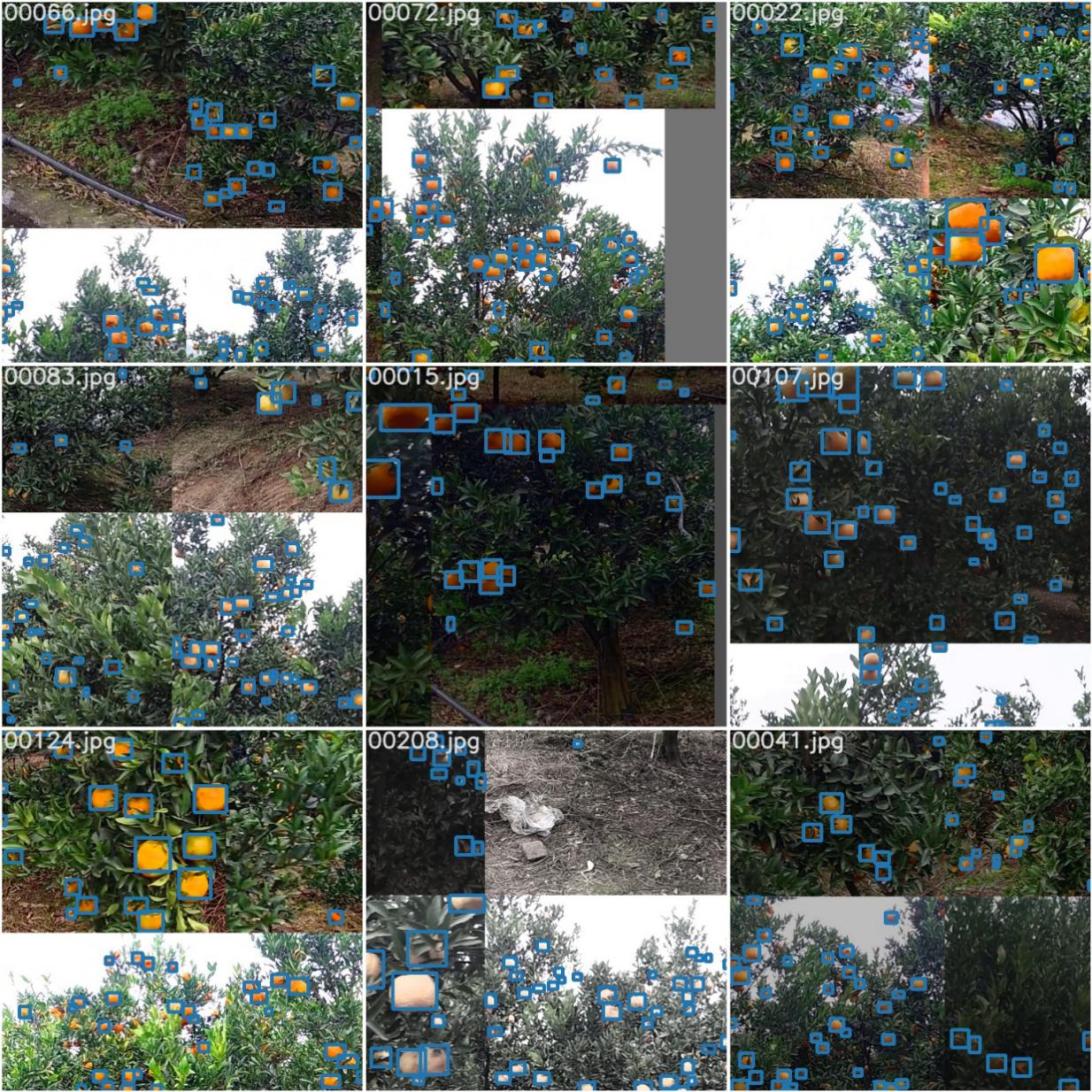


Appendix 2. Mosaic augmentation results


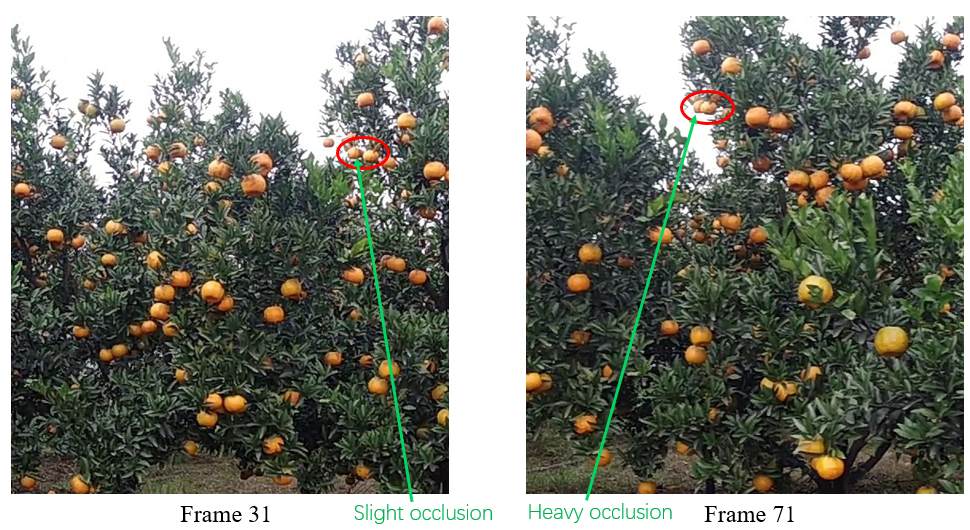


Appendix 3. Change in shading of the same fruit in the video sequence.

The green arrow shows fruit A in frame 31 almost side by side with another fruit B; in frame 71 most of fruit A has been obscured by fruit B. The pixel area of fruit A is gradually decreasing.


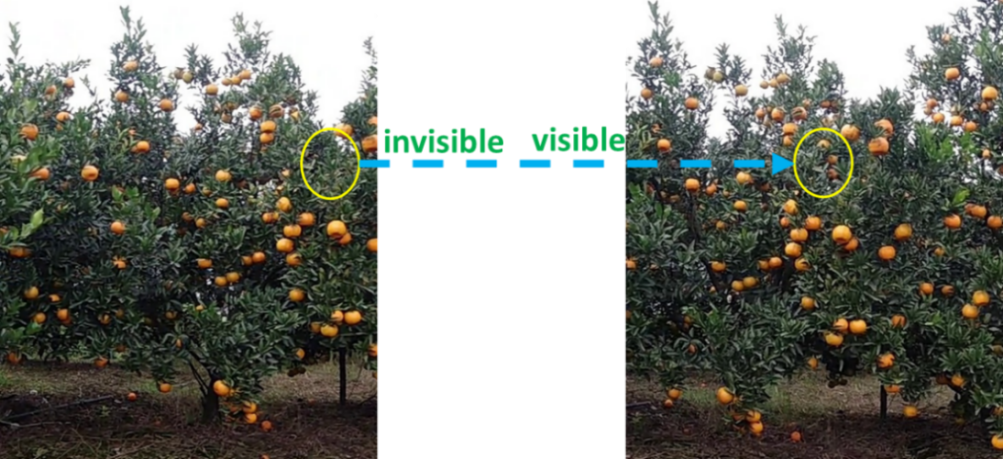


Frame 50 Frame 70

Appendix 4. Visible state of the fruit.

In the 50th frame, the marked yellow area enters the camera view, and the fruit in the area is completely occluded by the leaves. However, with the change in the acquisition perspective, the fruit in the yellow area in the 70th frame can be clearly observed.


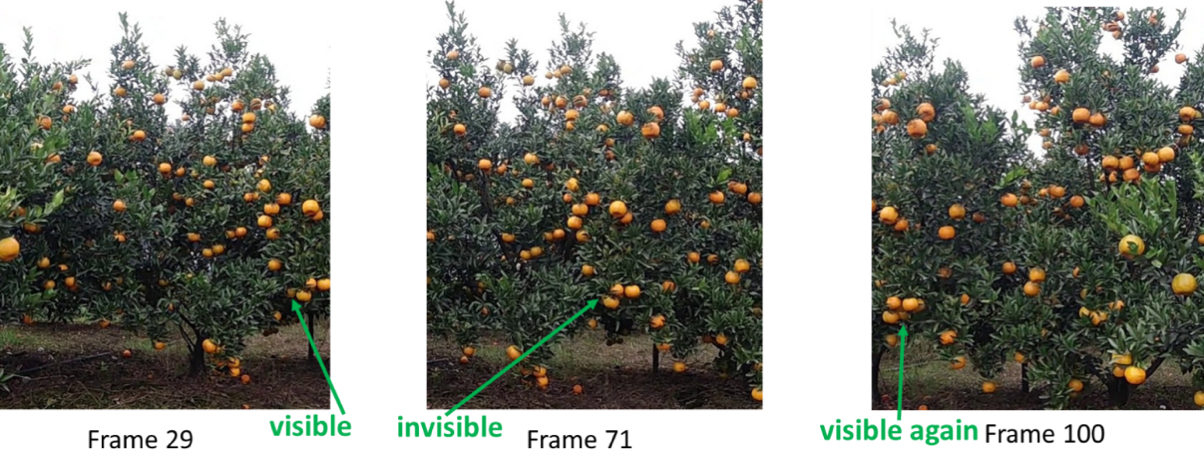


Appendix 5. Video sequence occlusion analysis. The orange indicated by the green arrow can be clearly observed in frame 29; however, as the acquisition device moves, the orange cannot be observed in frame 71 because it was occluded by an orange next to it. Furthermore, immediately before the fruit leaves the field of view of the camera, the fruit indicated by the green arrow appears clearly in frame 100 owing to the change in the acquisition viewpoint.


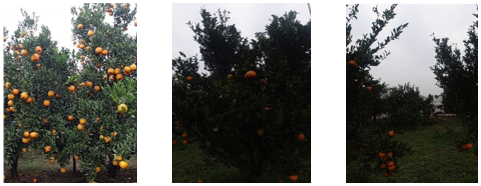


Sequence 1 Sequence 2 Sequence 3


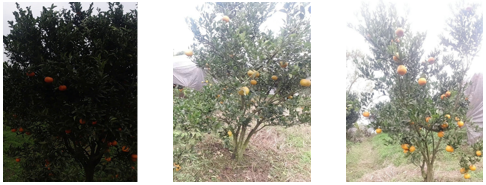


Sequence 4 Sequence 5 Sequence 6

Appendix 6. Video sequence display

The video sequences contain a variety of complex occlusions, scale variations, and different shooting conditions, which are frequently encountered in practical application. Video sequence 1 involves dense fruit growth, video sequences 2–4 are poorly lit, video sequence 5-6 is overexposed with bumps and dramatic camera shake during shooting.
